# The effect of different flushing methods in a short peripheral catheter^1^

**DOI:** 10.1590/s0102-865020190080000004

**Published:** 2019-10-14

**Authors:** Cuiling Tong, Xiaochun Peng, Hong Hu, Zongwen Wang, Hong Zhou

**Affiliations:** I Master, Yangtze University, School of Pathology Basic Medicine Faculty of Medicine, Hubei, P.R. China. Design of the study, acquisition and analysis of data, manuscript writing, final approval.; II PhD, Professor, Yangtze University, School of Pathology Basic Medicine Faculty of Medicine, Hubei, P.R. China. Intellectual contribution to the study, critical revision, final approval.; III Master, Yangtze University, School of Pathology Basic Medicine Faculty of Medicine, Hubei, P.R. China. Acquisition and analysis of data, final approval.; IV Master, Professor, Yangtze University, School of Pathology Basic Medicine Faculty of Medicine, Hubei, P.R. China. Design of the study, final approval.

**Keywords:** Catheterization, Peripheral, Blood Vessels, Rabbits

## Abstract

**Purpose:**

To develop a rabbit model of a short peripheral catheter (SPC) and to observe the effects of different flushing methods on blood vessels.

**Methods:**

Thirty rabbits were randomly divided into three groups (A, B, and C), with ten rabbits per group. In group A, we used pulsed flush; in group B, we used uniform flush; and no treatment was used in group C.

**Results:**

We observed that a uniform flush reduced blockage, phlebitis, and exudation compared to a pulsed flush by visual observation. The histopathological examination found that the morphological changes in group A were more severe than in group B and C related to loss of venous endothelial cells, inflammatory cell infiltration, edema, epidermal and chondrocyte degeneration, except for the thrombosis on group B that was more serious than in group A, especially in the distal side of puncture points. The distal region of groups A and B had more inflammatory cell infiltration than the proximal region. Thrombosis was more severe in the distal region than in the proximal region in group B.

**Conclusions:**

The uniform flush produced less damage to the vascular endothelium and surrounding tissues and was superior to the pulsed flush. However, the uniform flush is prone to thrombosis.

## Introduction

Short peripheral catheters (SPCs) are commonly used in the infusion of liquids, drugs, and blood products, and are needed by 70% of patients. SPCs are prone to complications such as blockage, phlebitis, and exudation, being necessary to replace them very often^1 - 2^ . Therefore, it is imperative to maintain a good stock of SPCs. Flushing is an effective treatment to keep SPC unobstructed. The methods most commonly used are slow uniform flush and pulsed flush^3 - 4^ . However, previous studies have different opinions on the effects of these two types of flushing methods. In vitro studies have shown that pulsed flush can remove solid deposits inside SPCs more effectively than the slow uniform flush. On the other hand, other studies found that the incidence of phlebitis in the slow uniform flush is lower than in the pulsed flush^4 - 6^ . The American practice standard for infusion therapy points the need to study the effects of pulsed flush^7^ . Therefore, this study describes an objective method to assess the effect of different flushing methods on blood vessels and provides microscopic evidence for the rationale of the flushing method selection.

## Methods

The study protocol was reviewed and approved by the Research Ethics Committee of Yangtze University.

Thirty male Japanese white rabbits were selected as experimental animals. They were provided by the Hubei College of Chinese Medicine (SCXK, 2014-0010, China). Each rabbit had a weight of 2.5~3.0 kg and was housed in individual cages inside the vivarium. The air temperature and humidity were maintained at 26±1 °C, 55±10 %, and 12 h light/dark cycles. The diet and activities of all the rabbits were normal. They stayed in acclimatization for one week.

###  Materials 

The materials used for the experiment were:

- A 24G short peripheral catheter SPC (Lingyan Medical Technology Co., Ltd., Suzhou, China);
- Pre-filled syringe (Weigao Group Medical Polymer Products Co. Ltd., Shandong, China);
- Saline solution;
- 3M dressing and adhesive plaster;
- Leica DM LB2 microscope (Imported from Germany),
- Hematoxylin and eosin staining solution (Zhuhai Besso Biotechnology Co., Ltd.).

###  Experimental process 

The 30 rabbits were divided into groups A, B, and C by a computer random number table, with ten animals in each group. First, the ear veins of rabbits in group A and B were identified, and the hair was cut off with medical scissors. After applying talcum powder, the residual hair was removed with a disposable skin knife, and then the skin was washed with saline solution. Second, the researcher fixed the rabbit’s ear base with one hand. This maneuver blocked the venous blood return and facilitated vein puncture. The researcher, with his other hand, pressed the rabbit’s body to prevent twisting. A second researcher pulled the rabbit ear and introduced the catheter. After the puncture was completed, the catheter was fixed with a 3M transparent applicator and medical tape. Finally, the rabbit ears were fixed with a self-made mesh elastic bandage earmuff to prevent SPC from falling off. The control group was labeled with a purple vein 3 cm from the ear tip of the rabbit, and no puncture was performed.

After the rabbit model was established, 5 mL pre-filled catheter irrigator (containing 0.9% sodium chloride injection with no preservative) was used for the tube. We compared the three methods to perform flushing in each group:

**Group A**: Used pulsed flush. The method consisted of 5 successive boluses, 1 mL flushed in 0.5 s each. The reference time of “flush-pause” sequence was 0.5 s flush, then 0.4 s pause (90 mL/min, flushing time is 9 s), until the end of the bolus^5^ .

**Group B**: Used uniform flush. The method was a single 5 mL bolus (10 mL/min, flushing time is 30 s), until the end of the bolus.

**Group C**: Without treatment/control group.

After flushing, the SPCs were adequately fixed and flushed once every 8 hours. Before flushing, the positive pressure connector was disinfected, and blood was withdrawn first.

**Figure 1:**
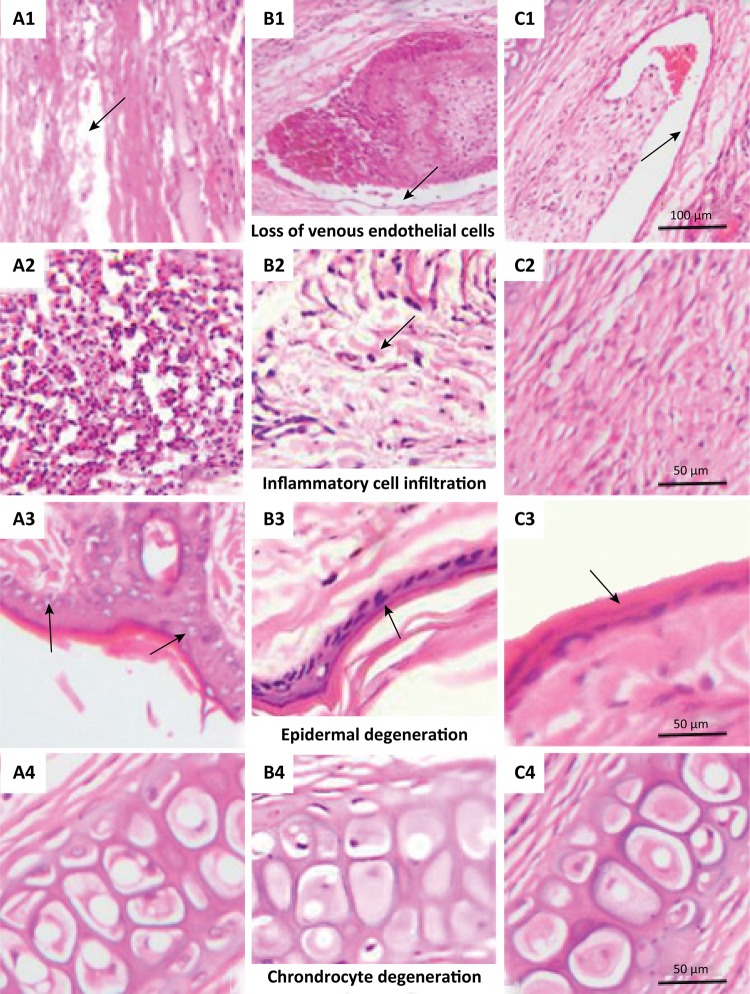
Regions of the ear vein specimen for histopathological examination. Regions of specimen in ear vein for histopathological examination. The extracted part was 2cm away from the point of puncture, wherein the first rectangular specimen of 0.5cm on the left side was the proximal region, and the second one of 0.5cm on the right side was the distal region.

The three groups were maintained for three days, and then euthanized by injecting 20 mL of air into the ear veins. The specimen range was 1.5~2 cm from the point of puncture, 0.5 cm (0.5×1 cm) on the vein sides (proximal region), and 2~2.5 cm from the point of puncture, 0.5 cm (0.5×1 cm) on the vein sides (distal region). A total of two samples were marked. This method is showed in Figure 1 . On the control group, the proximal and distal specimens were 2.5 cm to 3 cm from the tip of the ear, 0.5 cm (0.5 cm × 1 cm) on the vein sides, and 3 cm to 3.5 cm, 0.5 cm (0.5 cm × 1 cm) on the vein sides. The samples were fixated in 4% neutral formaldehyde immediately. The samples then underwent conventional dehydration, paraffin embedding, cross-section sectioning, hematoxylin-eosin (HE) staining, and finally observed by the pathologist, blind to the different groups.

###  Indexes for observation 

#### Visual observation

A) **Blockage**: No blockage meant blood could be withdrawn and liquid injected; partial blockage meant we could not pull back blood, but injection of liquids into the vein was possible; complete blockage meant blood could not be withdrawn nor liquid could not be injected into the vein^8^ .

B) **Phlebitis**: Using the standard practice of the American Infusion Nurses Society (INS) phlebitis grade was 0 to 4^7^ . Grade 0 is asymptomatic; in Grade 1 there is erythema at the puncture site; in Grade 2 erythema and/or edema appears at the puncture site; in Grade 3 there is erythema at the puncture site, forming strips/grains, and accessible to the venous cord; in Grade 4 there is a red spot at the puncture site, forming strips/grains, and accessible venous cord (length >2.5cm) with purulent liquid.

C) **Exudation**: It was based on the standard of the American INS, grading exudation from 0 to 4^7^ . Grade 0 is asymptomatic; in Grade 1 the skin is white, cold, and the edema <2.5cm; in Grade 2 the skin is white, cold, and the edema range is between 2.5cm and 5cm; in Grade 3 the skin is white, translucent, cold, and the edema range >5cm; in Grade 4 the skin is white, translucent, tight, and with exudation. The edema is discolored, bruised, swollen, and the edema range >5cm, with circulatory disorder^7^ .

#### HE stains

**A) Morphology**: respect the integrity of the vessel wall, the structure of the vascular endothelial cells, whether the blood vessels, surrounding connective tissue and the blood vessel wall adheres to inflammatory cells or not.

**B) Inflammatory cell count**: The inflammatory cells of each section were counted under high magnification (x400) using Image-Pro Plus software (6.0). Each specimen was recorded over ten sheets and then averaged.

C) **Histopathological grading**: The Kuwahara’s histopathological observation criteria were used to grade the findings, added chondrocyte degeneration was not included^9 , 11^ . The histopathological grading is shown in Table 1 .

**Table 1:** Criteria for histopathological examination.

Histopathological findings	Grade
**Loss of venous endothelial cells**	
None	0
Less than 1/3 of vein in cross-section	1
1/3-2/3 of vein in cross-section	2
More than 2/3 of vein in cross-section	3
**Inflammatory cell infiltration**	
None	0
Few inflammatory cells in venous wall or perivascular tissue	1
Many inflammatory cells in venous wall or perivascular tissue	2
More diffuse and denser inflammatory cells in perivascular tissue	3
**Edema**	
None	0
Localized in perivascular tissue	1
More diffuse edema	2
Edema throughout the whole area	3
**Thrombus**	
None	0
Less than 1/3 of vein in cross-section	1
1/3-2/3 of vein in cross-section	2
More than 2/3 of vein in cross-section	3
**Epidermal degeneration**	
None	0
Some degeneration epidermal cells near the vein	1
Numerous degeneration epidermal cells near the vein	2
More diffuse and severe epidermal degeneration	3
**Chondrocyte degeneration**	
None	0
Some chondrocyte degeneration	1
Numerous chondrocyte degeneration	2
More diffuse and severe chondrocyte degeneration	3

##  Statistical analysis 

Statistical calculations were performed using SPSS software version 22.0 (SPSS Inc., Chicago, IL, USA). The measured data of the normal distribution is represented by `x±s. Multiple groups were compared using analysis of variance (ANOVA), and the two groups (A and B) were compared using the t-test. Non-parametric tests were used for non-normal distribution, and H-test was used for multiple groups. The Rank sum test was used for pairwise comparison of the grade data. A *p* -value < 0.05 was considered statistically significant.

## Results

###  Visual observation 

No rabbit died during the experiment. One of the SPCs in the group A was completely blocked when the tube was maintained for 72h. The degree of occlusion, phlebitis, and exudation in group B were significantly lower than in group A. The difference between the two groups was statistically significant ( *p* < 0.05) ( Table 2 ).

**Table 2:** Comparison of the visual observations results of the three groups ( *n* =10).

Groups	Blockage			*Z*	*p*	Phlebitis				*Z*	*p*	Exudation				*Z*	*p*
		
	No	Partial	Complete			0	1	2	3			0	1	2	3		
A	6	3	1	-2.185	<0.05	1	4	4	1	-4.194	<0.01	2	6	2	0	-3.231	<0.01
B	7	3	0			10	0	0	0			9	1	0	0		

“ *n* ” means only the number of rabbits in each group.

###  Histopathological changes 

**Figure 2:**
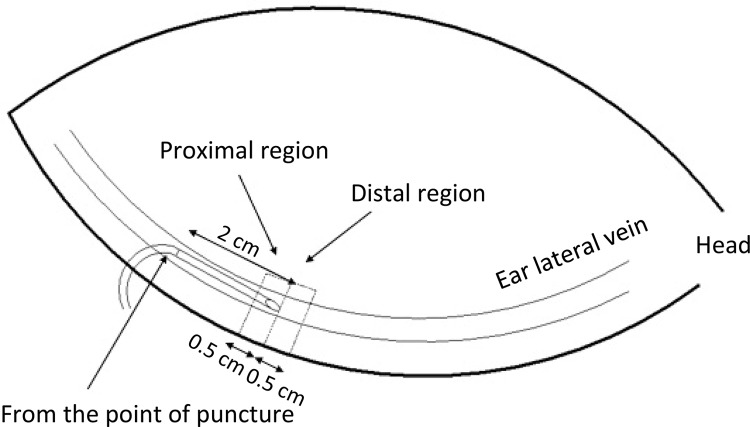
The image of pathological grading. A1, B1 and C1 show the effect of pulsed flush, uniform flush and no intervention on loss of venous endothelial cells respectively (Cross-section, 100 μm means magnification, x200), A2, B2 and C2 show the effect of pulsed flush, uniform flush and no intervention on inflammatory cell infiltration in the perivascular tissue (Cross-section, 50 μm means magnification, x400), A3, B3 and C3 show the effect of pulsed flush, uniform flush and no intervention on epidermal degeneration respectively (Cross-section, 50 μm means magnification, x400), also A4, B4 and C4 show the effect of pulsed flush, uniform flush and no intervention on Chondrocyte degeneration in the vein attachment (Cross-section, 50 μm means magnification, x400).

Figure 2 displays the typical image of an ear vein after different treatments. As could been seen from Figure 2 , in the group A, there was an obvious loss of venous endothelial cells (black arrow in A1), and there was an amount of inflammatory cell infiltration in the perivascular tissue (black arrow in A2), and an obvious vacuolar degeneration occurred in the epidermal cells (black arrow in A3), and little chondrocyte degeneration; the group B had a complete vascular wall structure, and the endothelial cells were visible on the inner wall of the vessel (black arrow in B1), also there was an amount of inflammatory cell infiltration in the perivascular tissue (black arrow in B2), epidermal cells were neatly arranged, less degenerated (black arrow in B3), there was little chondrocyte degeneration; vascular endothelial cells in the group C were intact (black arrow in C1), no obvious inflammatory cell infiltration was observed in the perivascular tissue, and the epidermal cells were arranged neatly (black arrow in C3), and also there was no degeneration of chondrocytes.

Table 3 summarizes the histopathological changes in the three groups after flushing. Group A experienced a severe loss of venous endothelial cells (Grades 1~3) in the distal part of the vein in all ( *n* =10) the animals. In group B, we observed a slight performance (Grade 1) in 5 out of 10 animals. At the proximal part of the vein, 8 out of 10 animals in group A had a moderate loss of venous endothelial cells (Grades 1~2), while 4 out of 10 animals in group B had slight changes (Grade 1). Also, group A experienced acute inflammatory cell infiltration (Grades 1~3) in the distal part of the vein in all the 10 animals, so as to group B (Grades 1~2). In the proximal part of the vein, all the 10 animals had inflammatory cell infiltration (Grades 1~2) in group A and B. Edema (Grades 1~3) was found in the distal part of the vein in 9 animals in group A (Grades 1~3), and also in 9 animals in group B (Grades 1~2). However, in the proximal part of the vein, the results of groups A and B had no statistical significance. In the distal part of the vein, a thrombus (Grades 1~3) was found in all the 10 animals in group B, but only in 8 animals in group A. In the proximal part of the vein, thrombus was found in 7 animals in group A and only in 4 animals in group B. At the same time, there was a severe epidermal degeneration (Grades 1~3) at the distal region in 9 out of 10 animals in group A and only in 2 out of 10 animals in group B (Grade 1), while at the proximal region in 5 animals in group A and 2 animals in group B. There was chondrocyte degeneration (Grades 1~2) at the distal region in all the 10 animals in group A and in 9 animals in group B, while at the proximal region, chondrocyte degeneration was present in 10 animals in group A and 9 animals in group B. In group C, we did not observe any changes.

**Table 3:** Comparison of pathological grades of rabbit ear veins in the three groups ( *n* =10).

Region	Group	Loss of venous endothelial cells				*Z*	*P*	Inflammatory cell infiltration				*Z*	*P*	Edema				*Z*	*P*
		
		0	1	2	3			0	1	2	3			0	1	2	3		
Distal	A ^a^	0	4	5	1	-2.500	<0.05	0	2	5	3	-2.409	<0.05	1	1	6	2	-2.127	<0.05
	B ^b^	5	5	0	0	-4.359	<0.01	0	7	3	0	-4.147	<0.01	1	7	2	0	-4.141	<0.01
	C ^c^	10	0	0	0	-4.091	<0.01	10	0	0	0	-4.082	<0.01	10	0	0	0	-4.104	<0.01
Proximal	A ^a^	2	5	3	0	-2.330	<0.05	0	5	5	0	-2.517	<0.05	0	4	5	1	-1.048	N.S.
	B ^b^	6	4	0	0	-4.119	<0.01	0	8	2	0	-4.194	<0.01	3	6	1	0	-4.108	<0.01
	C ^c^	10	0	0	0	-4.082	<0.01	10	0	0	0	-4.359	<0.01	10	0	0	0	-4.091	<0.01

**Region**	**Group**	**Thrombus**				***Z***	***P***	**Epidermal degeneration**				***Z***	***P***	**Chondrocyte degeneration**				***Z***	***P***
		
		**0**	**1**	**2**	**3**			**0**	**1**	**2**	**3**			**0**	**1**	**2**	**3**		

Distal	A ^a^	2	2	0	6	-2.387	<0.05	1	6	2	1	-2.865	<0.01	0	6	4	0	-1.757	N.S.
	B ^b^	0	1	1	8	-4.194	<0.01	8	2	0	0	-4.194	<0.01	1	5	4	0	-4.091	<0.01
	C ^c^	10	0	0	0	-4.119	<0.01	10	0	0	0	-4.104	<0.01	10	0	0	0	-4.119	<0.01
Proximal	A ^a^	3	2	0	5	-2.330	<0.05	4	3	2	1	-2.440	<0.05	0	5	5	0	-2.500	<0.05
	B ^b^	6	0	0	4	-4.119	<0.01	8	1	1	0	-4.194	<0.01	1	5	4	0	-4.091	<0.01
	C ^c^	10	0	0	0	-4.082	<0.01	10	0	0	0	-4.065	<0.01	10	0	0	0	-4.359	<0.01

“ *n* ” indicates the number of slices per group in different regions; “a” indicates that the *p* value of the rank is a comparison between group A and B; “b” indicates that the *p* value of the rank is a comparison between group B and the control group; “c” indicates that the *p* value of the rank is a comparison between the control group and the group A; Not Significant (NS) is represented by N.S. The numbers in the table indicate the number of occurrences at different levels. The comparison of H test between the three groups was *p* < 0.01.

###  Inflammatory cell count 

Light microscopy showed that the inflammatory cell count in the distal region was higher in group A than in group B ( *p* < 0.05). In the proximal region of the puncture site, there was no significant difference between groups A and B ( *p* > 0.05) ( Table 4 ).

**Table 4:** Comparison of the inflammatory cell count in the three groups.

Region	Groups	Number of inflammatory cells	*t*	*p*
Distal	A	202.20±104.36	2.972	<0.05
	B	100.60±28.15		
Proximal	A	116.00±32.07	3.255	N.S.
	B	75.00±23.61		

“ *n* ” indicates the number of slices per group in different regions.

## Discussion

Intravenous infusion is a standard procedure in clinical practice. The most common used catheters are SPC, midline catheter, peripherally inserted central catheter, central venous catheter, and implantable venous access port. Among them, SPC is the most commonly used catheter for infusion and blood transfusion. It is estimated that up to 85% of emergency patients require infusion therapy, and in most of them, we use SPC. The widespread use of SPC can bring complications, such as phlebitis, extravasation, and tube occlusion. Maintaining the catheter unobstructed and extending the catheter indwelling time has always been a problem. Flushing can maintain the patency of the catheter, but the results of the specific flushing methods are mixed. The INS guide recommended the pulsed flush, but its actual effect needs further verification by clinical research^7^ .

Current research of the pulsed flush speed was based on the research results by Guiffant *et al* .^3 , 5^ , but this was an in vitro study. In this study, our results support the notion that pulsed flush is more likely to cause venous injury than slow uniform flush. The results of this study showed that at the distal region, the pulsed flush was more likely to cause loss of venous endothelial cells, inflammatory cell infiltration, edema, and epidermal degeneration than the uniform flush ( *p* < 0.05). In the proximal region, the pulsed flush was more likely to cause a loss of venous endothelial cells, inflammatory cell infiltration, epidermal, and chondrocyte degeneration than the uniform flush ( *p* < 0.05). The pulsed flush made the venous blood turbulent, and the total shear force generated by the liquid flow was higher than the laminar shear force generated by one bolus of the uniform flush^5^ . The shearing force in the turbulent region caused the fluid flow to be unstable, which might origin shed and damage of the vascular endothelial cells, induce the release of inflammatory factors, adhere to the white blood cells in the blood, and aggravate the inflammatory reaction^5^ . Besides, pulsed flush rapidly increases and decreases local pressure in the blood vessel lumen, increases the permeability of the blood vessel wall, edema in the surrounding tissue, blood vessel dilation at the puncture site, blood exudation to the extravascular space, and makes the vessel prone to infection^11^ . The inflammatory cell count caused by pulsed flush was significantly higher than the uniform flush ( *p* < 0.05), as shown in Table 4 , which is consistent with the histopathological grading results in this study.

However, in the distal region, the thrombosis in group B was more severe than in group A ( *p* < 0.05). When the uniform flush was pushed, the sealing liquid flows to the tip of the SPC in a direct current flow. Under viscous resistance, the flow rate of the sealing liquid is the largest at the centerline of the blood vessel cavity, and the flow velocity gradually decreases in the blood vessel wall. As the blood flow rate slows, blood stagnates gradually, causing the structure of vascular endothelial cells to change. When the collagen fibers were exposed, the process of coagulation was initiated, thus increasing the chance of thrombosis. At the same time, the deposits adhering to the inner wall of the blood vessel are not easily removed and is easy to damage the vascular endothelial cells at the adhesion site, thereby inducing the formation of intravascular thromboses^14^ . When using pulsed flush, the sealing fluid flows to the tip of the SPC in a disordered turbulent flow, which can effectively remove the deposits adhering to the inner wall of the blood vessel, thereby reducing the stimulation and damage of the deposits on the inner wall of the blood vessel at the distal end of the puncture point, and reducing thrombosis^13 - 14^ . As shown in the results of this study, the uniform flush was more likely to form thrombus than the pulsed flush ( *p* < 0.05). However, the results were inconsistent with the visual observation that direct bolus catheter occlusion was lighter than the pulsed tube. The reasons may be thrombosis in a part of the blood vessel or part of the blood vessel cross-section. The granules of the liquid were small and still can flow through the narrow space without thrombus formation during rinsing. Another reason could be the difference in the judgment of the two observation indexes, and the degree of the uniform flush was lighter than the pulsed flush during visual observation, but in the pathological observation of uniform flush, we appreciated more serious thrombosis, indicating that visual observation was subjective, while pathological observation is more objective.

Guiffant *et al* .^5^ found that pulsed flush of the catheter lumen was more effective than the slow uniform flush (90 ± 3% *vs.* 79.1%). In in vitro experiments, Ferroni *et al* .^4^ showed that pulsed flush significantly reduced the number of bacteria in the catheter lumen compared to slow uniform flush. These results were inconsistent in our study. Although pulsed flush was observed to have a better flushing effect than slow uniform flush, no difference among flushing methods was observed for the vascular endothelium and surrounding tissue, which caused direct or indirect damage. Also, the speed of pulsed flushes, such as push 1 ml pause 0.4s, each push taking 0.5s, was about 90 mL/min. This was inspired by Guiffant’s and other studies^5 , 12^ . The speed of the slow uniform flush was slow and constant, which was less than 10 mL/min, according to our domestic standards^12^ . The speed was different from Guiffant’s study, and the results were also different. Jia’s research showed that the incidence of phlebitis in pulsed flush was higher than in slow uniform flush, and his results were consistent with this study^6^ . This indicates that the pulsed flush was not suitable for patients with poor vascular conditions such as diabetes and cardiovascular disease. The histopathological findings and visual observation of this study were consistent. The pulsed flush’s rate of blockage, phlebitis, and exudation were higher than those of the slow uniform flush ( *p* <0.05).

Some researchers found that chondrocytes have inflammatory infiltration and necrosis after using chemotherapy drugs^9 , 11^ . However, there was no significant difference in chondrocyte degeneration between groups A and B in the distal part of the vein in our study ( *p* > 0.05). This study selected physiological saline as the flushing fluid, instead of stimulating drug modeling, to better observe the influence of different flushing methods on blood vessels and avoid interference from other factors, thus ensuring the quality of the research.

There was no uniform standard for the speed of the uniform flush and the time of pulsed flushing. Although the recommended speed for the uniform flush in China was less than 10 mL/min, the amount of clinical research backing this recommendation is small and not convincing. Since this study did not cover the observation of the SPC time by different flushing methods, there are limitations. It is expected that more clinical studies would increase the comparison of different speeds at the edge tube. In addition to the subjective judgment of visual observation, they could increase the observation of blood vessels by cytological and imaging techniques.

## Conclusions

After the SPC was widely used in clinical practice, complications such as blockage and phlebitis appeared. The flush methods could effectively reduce those complications. In this study, it was found by visual and pathological morphology that slow uniform flush could reduce SPC damage to the vascular endothelium and surrounding tissues, even though it was prone to thrombosis. It is suggested that the clinically selectable flushing method could be based on the patient’s and vascular conditions.

## Footnotes

1 Research performed at the Department of Nursing, Pathology Medicine Experimental Center, Department of Medicine, Yangtze University, Jingzhou, China.
